# Treatments after Immune Checkpoint Inhibitors in Patients with dMMR/MSI Metastatic Colorectal Cancer

**DOI:** 10.3390/cancers14020406

**Published:** 2022-01-14

**Authors:** Quang Loc Bui, Léo Mas, Antoine Hollebecque, David Tougeron, Christelle de la Fouchardière, Thomas Pudlarz, Emily Alouani, Rosine Guimbaud, Julien Taieb, Thierry André, Raphaël Colle, Romain Cohen

**Affiliations:** 1 Department of Medical Oncology, Assistance Publique des Hôpitaux de Paris (AP-HP), Hôpital Saint-Antoine, Sorbonne Université, 75012 Paris, France; quangloc.bui.md@gmail.com (Q.L.B.); leo.mas@aphp.fr (L.M.); thomas.pudlarz@aphp.fr (T.P.); thierry.andre@aphp.fr (T.A.); raphael.colle@aphp.fr (R.C.); 2 The Nuclear Medicine and Oncology Center, Bach Mai Hospital, Hanoi 116300, Vietnam; 3 School of Medicine and Pharmacy, Vietnam National University, Hanoi 123105, Vietnam; 4 Drug Development Department (DITEP), Gustave Roussy, Saclay University of Paris, 94800 Villejuif, France; antoine.hollebecque@gustaveroussy.fr; 5 Department of Gastroenterology, Poitiers University Hospital, 86000 Poitiers, France; david.tougeron@chu-poitiers.fr; 6 Medical Oncology Department, Centre Leon Berard, Lyon I University, 69008 Lyon, France; christelle.delafouchardiere@lyon.unicancer.fr; 7 Digestive Medical Oncology Department, CHU Toulouse—IUCT Rangueil-Larrey, 31059 Toulouse, France; emilyalouani@yahoo.fr (E.A.); guimbaud.r@chu-toulouse.fr (R.G.); 8 Department of Digestive Oncology, Georges Pompidou European Hospital, Paris Descartes University, Sorbonne Paris Cité, 75004 Paris, France; julien.taieb@aphp.fr; 9 Centre de Recherche Saint-Antoine, Equipe Instabilité des Microsatellites et Cancer, Equipe Labellisée par la Ligue Nationale Contre le Cancer, INSERM Unité Mixte de Recherche Scientifique 938, Sorbonne Université, 75012 Paris, France

**Keywords:** metastatic colorectal cancer, microsatellite instability, mismatch repair deficiency, chemotherapy after immunotherapy

## Abstract

**Simple Summary:**

Several studies suggested an enhanced efficacy of conventional treatments (CT, i.e., chemotherapy +/− targeted therapy) administered after immune checkpoint inhibitors (ICI) in certain tumor types, but no data are available concerning metastatic colorectal cancer (mCRC) patients harboring mismatch repair deficiency/microsatellite instability (dMMR/MSI). The aim of our study was to assess the outcomes of dMMR/MSI mCRC patients receiving CT after ICI failure. We retrospectively collected clinical data from a multicentric cohort of 31 patients. Although limited by the small number of patients, our results did not suggest improved outcomes with CT in our population, and no significant association with previous ICI efficacy or with anti-VEGF agents was evidenced. However, prolonged disease control was observed in several cases, suggesting that some patients might derive an unexpected benefit from post-ICI treatments. With ICI becoming the standard of care in patients newly diagnosed with dMMR/MSI mCRC, these results might help to inform clinical decision-making and to guide future therapeutic strategies for these patients.

**Abstract:**

Background: Several studies reported improved outcomes with conventional treatments (CT, i.e., chemotherapy ± targeted therapy) administered after immune checkpoints inhibitors (ICI) in certain tumor types. No data are available concerning patients (pts) with metastatic colorectal cancer (mCRC) harboring mismatch repair deficiency/microsatellite instability (dMMR/MSI). We aimed to assess the outcomes of dMMR/MSI mCRC pts receiving CT after ICI failure. Methods: We conducted a retrospective multicenter study investigating the outcomes of all dMMR/MSI mCRC pts who received post-ICI CT between 2015 and 2020. Results: 31 pts (male 61%, median age 56 years) were included. ICI was an anti-PD(L)1 monotherapy in 71% of pts, and 61% received >2 lines before post-ICI CT. The overall response rate and disease control rate were 13% and 45%, with a median progression-free survival (PFS) and overall survival of 2.9 and 7.4 months, respectively. No association of the outcomes with either ICI efficacy or anti-angiogenic agents was observed. Prolonged PFS (range 16.1–21.3 months) was observed in 4 pts (13%). Conclusions: Although conducted on a limited number of patients, our results do not support an association of previous ICI treatment with an enhanced efficacy of CT in dMMR/MSI mCRC. However, prolonged disease control was observed in several cases, suggesting that some pts might derive an unexpected benefit from post-ICI treatments.

## 1. Introduction

Approximately 5% of metastatic colorectal cancers (mCRC) exhibit a deficient DNA mismatch repair (dMMR) system, responsible for a molecular phenotype known as microsatellite instability (MSI) [1]. In 80% of cases, this deficiency results from the epigenetic silencing of *MLH1* (sporadic cases), which is frequently associated with the *BRAF^V600E^* mutation (60%), whereas one case in five arises from a germline mutation in one of the MMR genes (Lynch syndrome) [1,2].

The dMMR/MSI status has emerged as a major predictive biomarker for the efficacy of immune checkpoint inhibitors (ICI), especially for mCRC patients [3]. ICI have demonstrated an impressive clinical activity amongst dMMR/MSI mCRC patients in several non-randomized phase II trials, with objective response rates varying from 30 to 60% and with durable clinical responses in heavily pretreated patients [3,4,5,6]. More recently, the KEYNOTE-177 phase III trial demonstrated the superiority of front-line pembrolizumab (anti-PD1 monoclonal antibody) in terms of progression-free survival (PFS) and quality of life over standard-of-care chemotherapy plus targeted therapy [7,8].

Despite high rates of response and a durable clinical benefit, around 50% of dMMR/MSI mCRC patients experience primary or secondary resistance to ICI [4,5,6,7]. With pembrolizumab becoming the standard of care for patients newly diagnosed with dMMR/MSI mCRC, the appropriate therapeutic strategy beyond progression becomes of particular interest. In *BRAF^V600E^*-mutated dMMR/MSI mCRC, the combination of encorafenib plus cetuximab is one therapeutic option, while patients with *BRAF^V600E^* wild-type dMMR/MSI mCRC are candidates for chemotherapy plus targeted therapy [9,10].

A growing body of evidence suggests an enhanced activity of conventional treatments (CT, i.e., chemotherapy ± targeted therapy) after exposure to ICI in other tumor types such as non-small cell lung cancer (NSCLC) or melanoma [11]. These observations argue for a potential synergistic effect of CT with previous ICI treatment that is hypothesized to rely on several mechanisms such as the positive immunomodulatory effects of chemotherapy and targeted therapies, sustained modifications of the tumor microenvironment after previous immune activation, or a long-lasting effect of ICI beyond progression [12].

However, dMMR/MSI mCRC represent a subset of tumors associated with distinct immune features for which the efficacy of CT after ICI failure has not been investigated [13,14]. Moreover, the chemosensitivity of dMMR/MSI mCRC has been subject to debate in the pre-ICI era. Several studies suggested a lower chemosensitivity, especially for 5FU, and worse survival of dMMR/MSI tumors compared to patients with CRC harboring a proficient MMR system (pMMR)/microsatellite stable status (MSS), while others did not [1,15,16,17,18,19]. A recent study suggested a benefit to adding anti-EGFR to chemotherapy in familial dMMR/MSI mCRC in the first-line setting [20].

In this study, we evaluated the efficacy of CT administered after ICI failure in a cohort of dMMR/MSI mCRC patients.

## 2. Materials and Methods

### 2.1. Study Population

We conducted a retrospective multicenter study in 6 French hospitals. All patients with dMMR/MSI mCRC who received CT beyond ICI, regardless of the reason of ICI discontinuation, were included. The dMMR/MSI status was determined by immunohistochemistry of the four MMR proteins (MLH1, PMS2, MSH6, and MSH2) and/or Polymerase Chain Reaction (Pentaplex panel), as previously described [21]. Tumors with a loss of expression of MLH1 and PMS2 harboring the *BRAF^V600E^* mutation and/or hypermethylation of the *MLH1* promoter were considered as sporadic. Tumors with *BRAF* wild-type and unmethylated *MLH1* or a loss of MSH2, MSH6, and PMS2 protein expression were considered as associated with Lynch syndrome.

### 2.2. Outcomes

The endpoints were the objective response rate (ORR), disease control rate (DCR), progression-free survival (PFS), and overall survival (OS) under post-ICI CT.

ORR was defined as the proportion of patients with a complete (CR) or partial radiological response (PR) as the best response, assessed by the local investigators according to the RECIST version 1.1 (Response Evaluation Criteria in Solid Tumors). DCR was defined as the ratio of patients with CR, PR, or stable disease as the best response according to RECIST 1.1, to the number of treated patients. PFS was calculated from the start of the first treatment after ICI to disease progression or death due to any cause, whichever occurred first. OS was calculated from the initiation of the first treatment after ICI to death from any cause.

### 2.3. Statistical Analysis

The median value (range) and frequency (percentage) were generated for the description of continuous and categorical variables, respectively. Survival curves were estimated using the Kaplan–Meier method and compared with a Log-rank test. *p* values of less than 0.05 were considered statistically significant, and all tests were two-sided. All analyses were performed using R software version 3.6.1.

## 3. Results

### 3.1. Patients’ Characteristics

Thirty-one dMMR/MSI mCRC patients were included from June 2015 to January 2020. The dMMR/MSI status was determined both by immunohistochemistry and PCR in 29 patients (94%), and by immunohistochemistry alone in two patients. Patients’ characteristics are presented in Table 1.

At the initiation of post-ICI therapy, the median age was 56 years, and 58% of patients had ECOG 0-1. Most patients (84%) had two or more metastatic sites, the most frequent being lymph nodes (77%), peritoneum (65%), and liver (52%). Mutations of *RAS* or *BRAF^V600E^* were each present in 26% of patients, and one patient harbored both *RAS* and *BRAF^V600E^* mutations. Overall, 39% and 29% of patients had a confirmed Lynch syndrome and sporadic origin, respectively.

Before ICI therapy, 48% of patients received two or more chemotherapy regimens ± targeted therapy (range 1–4), and 100%, 94%, and 68% had been exposed to fluoropyridines, oxaliplatin, and irinotecan, respectively. The median PFS on the last chemotherapy administered before ICI was 3.7 months (95% CI [2.66–8.36]), with a DCR and an ORR of 52% and 16%, respectively.

ICI therapy was an anti-PD(L)1 monotherapy for 71% of patients (anti-PD1 *n* = 15, anti-PDL1 *n* = 7) and a combination for nine patients (Table 1). All but five patients treated with an anti-PD1 monoclonal antibody (16%) received ICI in a clinical trial. The median time of ICI treatment was 5.6 months (95% CI [3.5–10.9]), leading to a DCR and an ORR of 65% and 16%, respectively. Progressive disease (PD) was the reason for ICI discontinuation in all except for three patients (10%) with severe immune-related adverse events and secondary PD.

The main regimens for post-ICI chemotherapy were FOLFOX (29%), FOLFIRI (29%), or trifluridine-tipiracil (16%), associated with an anti-vascular endothelial growth factor (VEGF) or anti-epidermal growth factor receptor (EGFR) monoclonal antibody in 39% and 19%, respectively (Table 2). Out of the patients receiving anti-VEGF- or anti-EGFR-containing regimens, 55% and 17% had previous PD with a regimen including that same molecule.

### 3.2. Efficacy

**Figure 1:**
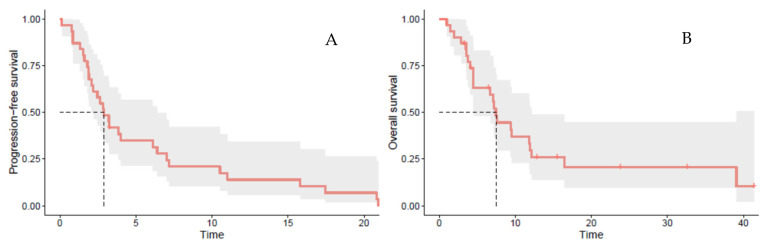
(**A**) Progression-free survival and (**B**) overall survival with chemotherapy ± targeted therapy post immune checkpoint inhibitor(s).

At the time of analysis, the median follow-up from the start of post-ICI CT was 23.8 months. PR or SD were achieved in four (13%) and 10 (32%) patients, resulting in a DCR of 45% (Table 2). The median PFS and OS were 2.9 months (95% CI [2.07–6.39]) and 7.4 months (95% CI [4.49–12.2]), respectively (Figure 1A,B).

Patients with progressive disease as the best response to post-ICI CT had a poorer ECOG performance status (*p* = 0.036) and tended to present with a more advanced age, more female patients, and more metastatic sites (Supplementary Table S1). No difference was observed according to the mutational status (*KRAS* and/or *BRAF* mutations), the type of and response to ICI, or the number of previous lines of therapy (Supplementary Table S1). In the univariate analysis (Table 3), ECOG ≥ 2 was associated with worse PFS (*p* = 0.045). No significant association of PFS and OS was observed with either the previous response to ICI or the anti-VEGF-containing post-ICI CT regimen. The median PFS and OS of patients who previously received ICI for more than six months (*n* = 15) were 6.1 months [2.1–17.4] and 9.4 months [7.4–NA] when compared to 2.8 months [1.6–7.0] and 4.5 months [3.8–NA] in patients treated for less than six months with ICI (*p* = 0.17 and 0.11 for PFS and OS, respectively) (Supplementary Figure S1).

Disease control lasting more than 12 months (range 16.1 to 21.3 months) was achieved in four patients (13%) receiving trifluridine-tipiracil (*n* = 2), FOLFIRI, and FOLFIRI3 + bevacizumab (the latter patient previously progressed under a bevacizumab-containing regimen). All four patients had *KRAS* wild-type tumors, and two harbored a *BRAF* mutation. Two achieved a partial response under post-ICI CT, and two had a durable stable disease. The details of the consecutive treatments and outcomes of these patients are presented in Table 4.

Under the last chemotherapy regimen before ICI, one of these patients was treated with FOLFIRI plus bevacizumab and achieved a PR with a PFS of 20 months, while the other three had an early progressive disease (PFS of 2.7 to 3.3 months). These same three patients experienced a disease stabilization under ICI with a treatment duration ranging from 5.6 to 19.5 months, including one in which ICI was discontinued due to toxicity. At the time of analysis, all four patients progressed under post-ICI CT; three of these patients were alive with a follow-up of 24.2 to 42.1 months after initiation of post-ICI CT.

## 4. Discussion

To our knowledge, our study is the first to assess survival outcomes associated with CT in patients with dMMR/MSI mCRC after treatment failure with ICI. We observed a median PFS of 2.9 months with a DCR and ORR of 45% and 13%, respectively, which are numerically close to the outcomes under the last CT before ICI (median PFS 3.7 months, DCR and ORR of 52% and 16%, respectively). Although based on hardly comparable populations, these results tend to compare favorably with those reported in phase III trials of mCRC beyond the second line of treatment [22,23]. However, in a large cohort of unselected dMMR/MSI mCRC patients, the median PFS (3.6 months) and OS (13.7 months) of patients receiving third-line chemotherapy ± targeted therapy were higher than those observed in our cohort, arguing against an enhanced efficacy of post-ICI chemotherapy in our population [16].

A growing amount of data, mostly derived from retrospective analysis and case series, suggests an enhanced efficacy of chemotherapy administered after ICI in other tumor types such as NSCLC, melanoma, or gastroesophageal adenocarcinomas (GEA) [11]. Additionally, one prior work retrospectively evaluated the outcomes of 29 mCRC patients, mostly with pMMR tumors (86%), treated with salvage chemotherapy ± targeted therapy after ICI [24]. An interesting DCR and ORR of 62% and 19%, respectively, were observed in a heavily pretreated population, but no difference was observed when comparing outcomes before or after ICI exposure in these patients [24]. However, none of these studies specifically evaluated the outcomes of dMMR/MSI patients, for which a distinct tumor biology and immune features might explain our results.

Still, our data suggest that a small amount of patients may derive an unexpected survival benefit from post-ICI anti-cancer treatments. A notably long-lasting disease control was achieved in four patients (13%) in our cohort, reaching a PFS of 16.1 to 21.3 months under post-ICI CT. Such prolonged disease controls were not observed among mCRC patients in the aforementioned study by Martin–Romano et al. [24], with a maximum PFS of 12 months. Interestingly, three of these long responders had an early progression on a previous chemotherapy before ICI, suggesting that some patients might still benefit from chemotherapy ± targeted therapy beyond ICI progression.

No clinical or molecular parameter was associated with patients’ outcomes in our study, except for the ECOG performance status, which is largely known as a major prognostic factor in heavily pretreated patients. Importantly, molecular parameters such as *RAS* and *BRAF* mutations were not associated with survival outcomes, but the heterogeneity of our population may have influenced this result.

A previous response to ICI was suggested as a potential predictive factor for subsequent chemotherapy efficacy in two studies [25,26]. No such association reached statistical significance in our population, but a trend toward an improved PFS (6.1 vs. 2.8 months, *p* = 0.17) and OS (9.4 vs. 4.5 months, *p* = 0.11) was observed in patients who previously received ICI for more than six months (Supplementary Figure S1). Moreover, three of the four long-responders in our cohort achieved disease control under a previous ICI treatment, suggesting that previous ICI efficacy might correlate with improved outcomes in a subset of patients.

Additionally, several studies reporting favorable outcomes with post-ICI chemotherapy focused on anti-VEGF-containing regimens, which could be related to the well-described immune-modulating effects of anti-angiogenic agents [27,28]. Notably, the association of paclitaxel and ramucirumab, an anti-VEGFR2 monoclonal antibody, in metastatic GEA previously exposed to ICI led to superior response rates compared to ICI-naïve patients (57.9% vs. 17.7%), as well as prolonged PFS (8.9 vs. 4.9 months; HR 0.37) and OS (15.0 vs. 7.6 months; HR 0.23) [29]. Similar results were reported with taxane plus ramucirumab following ICI exposure in two other studies of GEA patients, while no difference was observed with other regimens such as taxane monotherapy or irinotecan [30,31]. A trend toward better outcomes in patients receiving antiangiogenic agents as part of their salvage chemotherapy regimen was also observed in mCRC patients [24]. However, no such association was observed in our study, with only one in 12 patients receiving an anti-VEGF monoclonal antibody, achieving PR and a DCR of 42%. To explain these contradicting results, we could hypothesize that the distinct tumor biology of dMMR/MSI mCRC is associated with specific resistance pathways that might not be overcome by anti-angiogenics agents.

Altogether, our results could indicate that, unlike other tumor types, chemotherapy and/or antiangiogenic agents might not be the preferred candidates to associate with anti-PD(L)1 monoclonal antibodies in dMMR/MSI mCRC, for which combinations with anti-cytotoxic T lymphocyte antigen 4 (CTLA4) antibodies or other molecules under development targeting putative immune checkpoints (such as LAG3, TIM3, TIGIT …) might represent a more promising approach for the future.

Our study has several limitations, including its retrospective nature and the small sample size that hampers any conclusions. Notably, no multivariate analysis was performed given the small sample size. Besides, the heterogeneity of our population (ECOG performance status, mutational status, number of prior treatment lines, type of post-ICI treatment) may have impacted our analyses and restricted the possibility to analyze any association of distinct regimens with patients’ outcomes. Further larger studies are needed to compare distinct treatment regimens after ICI exposure. Nevertheless, this is the first study to evaluate the efficacy of chemotherapy ± targeted therapy after ICI in dMMR/MSI mCRCs.

## 5. Conclusions

In conclusion, our results, although limited by the small number of patients, do not support an association of a previous ICI treatment with an enhanced efficacy of chemotherapy ± targeted therapy in dMMR/MSI mCRC. However, a notable long-lasting disease control and prolonged survival were observed in several cases, suggesting that ICI might positively influence outcomes on subsequent treatments in a subset of patients. Further studies are needed to more precisely evaluate the outcomes of these patients and explore the tumor immune microenvironment of tumors with long-lasting disease control with chemotherapy ± targeted therapy after ICI in dMMR/MSI mCRCs.

## Figures and Tables

**Table 1:** Baseline characteristics.

		N (%)
Number of patients		31 (100)
Median age (range)		56 (28–77)
Gender	Male	19 (61)
	Female	12 (39)
ECOG	0–1	18 (58)
	≥2	13 (42)
Metastatic sites	1	5 (16)
	≥2	26 (84)
Mutational status	RAS/BRAF wild-type	14 (45)
	RAS-mutated	8 (26)
	BRAF-mutated	8 (26)
	RAS- and BRAF-mutated	1 (3)
Mechanism of MMR deficiency *	Lynch syndrome	12 (39)
	Sporadic	9 (29)
	Unknown	10 (32)
Number of treatment lines prior to ICI	1	16 (52)
	≥2	15 (48)
Exposure prior to ICI	Fluoropyrimidine	31 (100)
	Oxaliplatin	29 (94)
	Irinotecan	21 (68)
	Anti-VEGF	16 (52)
	Anti-EGFR	11 (36)
Type of immunotherapy	Anti-PD1 monotherapy	15 (48)
	Anti-PDL1 monotherapy	7 (23)
	Anti-PD(L)1 + anti-CTLA4	5 (16)
	Anti-PD(L)1 + Others **	4 (13)
Best response to ICI	Progressive Disease	11 (36)
	Stable Disease	15 (48)
	Partial Response	5 (16)
Reason for ICI discontinuation	Progressive disease	28 (90%)
	Toxicity	3 (10%)

* Based on BRAF mutational status, MLH1 methylation status, and MMR protein expression pattern; ** Others: Inducible T-cell COStimulator (ICOS) targeted therapy *n* = 1, OX40 agonist *n* = 1, anti T cell immunoglobulin and mucin domain-containing protein 3 (TIM3) *n* = 1, pexidartinib *n* = 1; MMR: Mismatch Repair; ICI: Immune Checkpoint Inhibitor(s); VEGF: Vascular endothelial growth factor; EGFR: Epidermal growth factor receptor.

**Table 2:** Characteristics of and outcomes with post-Immune Checkpoint Inhibitor(s) chemotherapy ± targeted therapy.

		N (%)
Number of prior therapy lines (Including Immune Checkpoint Inhibitor(s))	2	12 (39)
	3	15 (48)
	4	3 (10)
	5	1 (3)
Chemotherapy regimen	5FU/Capecitabine monotherapy	2 (6)
	Trifluridine-tipiracil	5 (16)
	FOLFIRI	9 (29)
	FOLFOX	9 (29)
	FOLFIRINOX	2 (6)
	Others *	4 (13)
Associated targeted therapy	No	13 (42)
	Bevacizumab or aflibercept	12 (39)
	Cetuximab or panitumumab	6 (19)
Best response	Progressive Disease	17 (55)
	Stable Disease	10 (32)
	Partial Response	4 (13)

* Others: 5FU + mitomycine *n* = 1, regorafenib *n* = 1, trastuzumab + lapatinib *n* = 1, panitumumab monotherapy *n* = 1.

**Table 3:** Univariate analysis of PFS and OS with post-Immune Checkpoint Inhibitor(s) therapy.

	Progression-Free Survival			Overall Survival		
	HR ^1^	95% CI ^1^	*p*-Value	HR ^1^	95% CI ^1^	*p*-Value
Age	1.02	0.99, 1.04	0.2	1.02	0.99, 1.05	0.12
Gender			0.4			0.6
Male	-	-		-	-	
Female	1.43	0.68, 3.01		1.26	0.55, 2.89	
ECOG			0.045			0.2
0–1	-	-		-	-	
2 or more	2.22	1.03, 4.77		1.76	0.75, 4.09	
Pathological type			0.4			0.8
Conventional adenocarcinoma	-	-		-	-	
Mucinous adenocarcinoma	0.73	0.34, 1.59		0.92	0.40, 2.14	
KRAS mutational status			0.3			0.6
Wild-type	-	-		-	-	
Mutated	1.65	0.68, 3.97		1.27	0.51, 3.13	
ICI best response			0.6			>0.9
Progressive disease	-	-		-	-	
Partial response or stable disease	0.83	0.37, 1.84		1.03	0.42, 2.52	
ICI duration			0.2			0.11
<6 months	-	-		-	-	
>6 months	0.60	0.28, 1.27		0.51	0.22, 1.18	
Post-ICI treatment line			>0.9			0.2
Third	-	-		-	-	
Fourth or more	1.01	0.47, 2.17		1.78	0.70, 4.55	
Post-ICI anti-VEGF			>0.9			0.8
None	-	-		-	-	
mAb anti-VEGF	1.01	0.48, 2.13		1.11	0.48, 2.53	

^1^ HR = Hazard Ratio, CI = Confidence Interval. Anti-VEGF: Anti-vascular endothelial growth factor; ICI = Immune Checkpoint Inhibitor(s).

**Table 4:** Treatment regimens and outcomes of patients with long-lasting disease control (>12 months) on post-Immune Checkpoint Inhibitor(s) chemotherapy ± targeted therapy.

Patient	Mutational Status	Pre-ICI Regimen	Line	Pre-ICI Best Response	Pre-ICI PFS	ICI	ICI Best Response	ICI Duration	Post-ICI Regimen	Line *	Post-ICI Best Response	Post-ICI PFS	OS
S005	*KRAS ^wt^ BRAF ^mut^*	FOLFOX	1	SD	3	Anti-PD1	SD	14.6	FOLFIRI	3	PR	17.7	24.2 ^+^
S013	*KRAS ^wt^ BRAF ^mut^*	Dabrafenib Trametinib Panitumumab	2	SD	2.7	Anti-PD1	SD	5.6	FOLFIRI 3 bevacizumab	4	SD	21.2	39.7
G004	*KRAS ^wt^ BRAF ^wt^*	FOLFOX Cetuximab	4	PD	3.3	Anti-PDL1	SD	19.5	Trifluridine -tipiracil	6	SD	21.3	33.2 ^+^
G006	*KRAS ^wt^ BRAF ^wt^*	FOLFIRI Bevacizumab	1	PR	20	Anti-PD1 + Other **	PD	6.6	Trifluridine -tipiracil	3	PR	16.1	42.1 ^+^

* Including ICI; ** Inducible T-cell COStimulator (ICOS) targeted therapy; ^+^ Alive at the time of analysis; *^wt^*: wild-type; *^mut^*: mutated; ICI: immune checkpoint inhibitors; PFS: progression-free survival; OS: overall survival; SD: stable disease; PR: partial response; PD: progressive disease.

## Data Availability

Data are available upon reasonable request.

## Supplementary Materials

The following supporting information can be downloaded at: https://www.mdpi.com/article/10.3390/cancers14020406/s1, Figure S1: Progression-free survival (A) and overall survival (B) with chemotherapy ± targeted therapy according to the duration of immune checkpoint inhibitor(s) treatment. Table S1: Patients’ characteristics according to the best response to post Immune Checkpoints Inhibitor chemotherapy +/− targeted therapy.
