# Supplementary material for: Treatments after Immune Checkpoint Inhibitors in Patients with dMMR/MSI Metastatic Colorectal Cancer
Source: Cancers (Basel). 2022 Jan 14;14(2):406. doi: 10.3390/cancers14020406 (PMC8774125; doi:10.3390/cancers14020406)

Treatments after immune checkpoint inhibitors in patients with dMMR/MSI metastatic colorectal cancer.

Supplementary material

Table S1. Patients' characteristics according to the best response to post Immune Checkpoints Inhibitor chemotherapy +/- targeted therapy.

	Best response to post ICI chemotherapy +/- targeted therapy		
**Characteristic**[1]	**Progressive disease**, N = 17[2]	**Partial response or stable disease**, N = 14[2]	**p-value**[3]
Age	58 (48, 64)	50 (37, 65)	0.3
Gender			0.3
Male	9 (53%)	10 (71%)	
Female	8 (47%)	4 (29%)	
ECOG			0.036
0-1	7 (41%)	11 (79%)	
2 or more	10 (59%)	3 (21%)	
Pathologic subtype			0.8
Conventionnal adenocarcinoma	9 (53%)	8 (57%)	
Mucinous adenocarcinoma	8 (47%)	6 (43%)	
Number of metastatic sites			0.2
1-2	7 (41%)	9 (64%)	
3 or more	10 (59%)	5 (36%)	
Mutational status			0.7
KRASwt/BRAFwt	8 (47%)	6 (43%)	
KRASm/BRAFwt	5 (29%)	3 (21%)	
KRASwt/BRAFm	3 (18%)	5 (36%)	
KRASm/BRAFm	1 (5.9%)	0 (0%)	
Mechanism of MMR deficiency*			0.4

Characteristic[1]	Best response to post ICI chemotherapy +/- targeted therapy<br>**Progressive disease**, N = 17[2]	 **Partial response or stable disease**, N = 14[2]	p-value[3]
Sporadic	5 (56%)	4 (33%)	
Lynch Syndrome	4 (44%)	8 (67%)	
Unknown	8	2	
ICI regimen			0.4
Anti-PD1 monotherapy	9 (53%)	6 (43%)	
Anti-PDL1 monotherapy	5 (29%)	2 (14%)	
Anti-PD(L)1 + Other**	3 (18%)	6 (43%)	
Duration of ICI treatment			0.4
<6months	10 (59%)	6 (43%)	
>6months	7 (41%)	8 (57%)	
Best response to ICI			>0.9
Progressive disease	6 (35%)	5 (36%)	
Partial response or stable disease	11 (65%)	9 (64%)	
Post-ICI CT line			0.7
Third	6 (35%)	6 (43%)	
Fourth or more	11 (65%)	8 (57%)	
Post-ICI anti-VEGF			0.8
None	10 (59%)	9 (64%)	
mAb anti-VEGF	7 (41%)	5 (36%)	

[1] *Based on BRAF mutational status, MLH1 methylation status and MMR protein expression pat-tern; **Others: Inducible T-cell COStimulator (ICOS) targeted therapy n=1, OX40 agonist n=1, anti T cell immunoglobulin and mucin domain-containing protein 3 (TIM3) n=1, pexidartinib n=1; MMR: Mismatch Repair; ICI: Immune Checkpoint Inhibitor(s); VEGF: Vascular endothelial growth factor

[2] Median (IQR); n (%)

[3] Wilcoxon rank sum test; Pearson's Chi-squared test; Fisher's exact test

Figure S1. Progression-free survival (A) and overall survival (B) with chemotherapy ± targeted therapy according to the duration of immune checkpoint inhibitor(s) treatment.

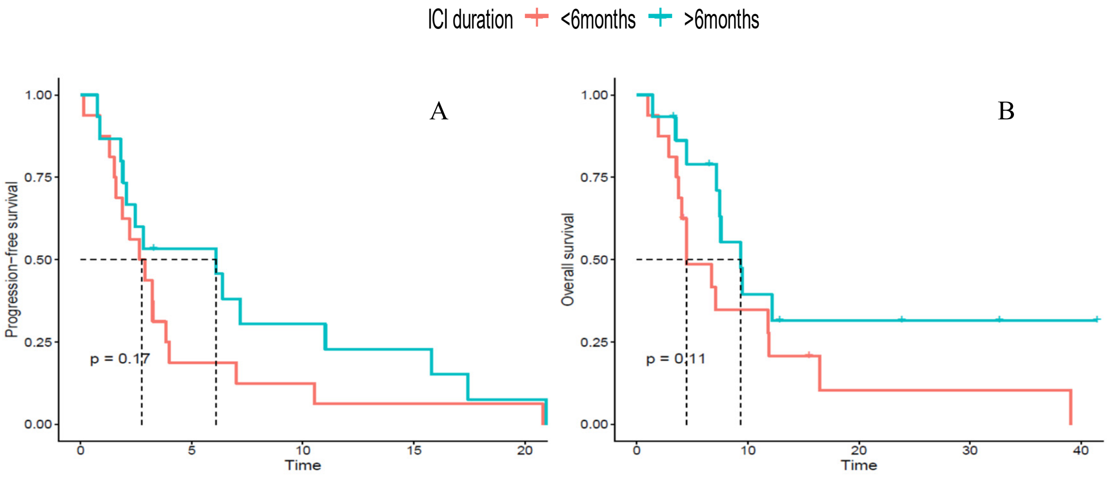

Supplement: Supplementary file 1 [file cancers-14-00406-s001.zip › cancers-1546280-supplementary.pdf]
